# Contribution of a Novel TetR/AcrR Family Transcriptional Regulator RalT of *Ralstonia pseudosolanacearum* Strain OE1‐1 to the Fine‐Tuning of Its Virulence

**DOI:** 10.1002/mbo3.70229

**Published:** 2026-02-22

**Authors:** Tatsuya Ueyama, Masayuki Tsuzuki, Sora Tateda, Yuki Terazawa, Aoi Ikeuchi, Akinori Kiba, Kouhei Ohnishi, Yasufumi Hikichi

**Affiliations:** ^1^ Faculty of Agriculture and Marine Science Kochi University Nankoku Japan

**Keywords:** quorum sensing, ralfuranone‐mediated feedback regulation, Ralstonia pseudosolanacearum, RalT

## Abstract

During the quorum‐sensing (QS)‐active state, the Gram‐negative phytopathogenic *Ralstonia pseudosolanacearum* strain OE1‐1 activates the transcriptional regulator PhcA, regulating the QS‐dependent genes including ralfuranone production‐related genes such as *ralA* and major exopolysaccharide EPS I production‐related genes such as *xpsR* encoding the transcriptional regulator XpsR, which are responsible for OE1‐1 virulence. Ralfuranone affects the regulation of more than 80% (QS/Ral‐dependent genes) of QS‐dependent genes, indicating the ralfuranone‐mediated feedback regulation of QS. To elucidate the mechanisms underlying the regulation of QS/Ral‐dependent genes, we analyzed the transcriptomes of *phcA*‐deletion (Δ*phcA*) and ralfuranone‐deficient (Δ*ralA*) mutants, as well as strain OE1‐1 by RNA‐sequencing. We found a novel TetR/AcrR family transcriptional regulator (RalT)‐encoding gene (*ralT*); the expression level of *ralT* reduced significantly in Δ*ralA* but not Δ*phcA* relative to expression level in strain OE1‐1, and RalT negatively regulated *ralT*. A transcriptome analysis of the *ralT‐*deletion mutant (Δ*ralT*) showed that the *ralT*‐deletion reduced the expression levels of 89.4% of positively QS/Ral‐dependent genes including *ralA* and *xpsR*, while enhanced 44.6% of negatively QS/Ral‐dependent genes. The transcript levels of these genes were positively correlated between Δ*ralT* and Δ*phcA* or Δ*ralA*, suggesting contribution of RalT to the regulation of some QS/Ral‐dependent genes. However, the *ralT*‐deletion enhanced EPS I production, suggesting that RalT represses the XpsR‐independent factor(s), which is regulated PhcA and contributes to the EPS I production. Furthermore, Δ*ralT* exhibited enhanced virulence, compared with strain OE1‐1. Collectively, results of the present genetic study suggest that RalT contributes to the exquisite fine‐tuning of OE1‐1 virulence.

## Graphical abstract

Predicted regulation of quorum sensing‐, ralfuranone‐, and RalT‐dependent genes (QS/Ral/p0599‐dependent genes) via a novel TetR/AcrR family transcriptional regulator RalT in *Ralstonia pseudosolanacearum* strain OE1‐1 was shown here. The regulation of *ralT* is dependent on ralfuranone, and contribution of RalT is popular with the regulation of some quorum sensing and ralfuranone‐dependent genes (QS/Ral/p0599‐dependent genes). Furthermore, ralfuranone biosynthesis‐related genes are regulated via positive feedback through RalT, suggesting that the regulation of QS/Ral/p0599‐dependent genes is exquisitely fine‐tuned through RalT.

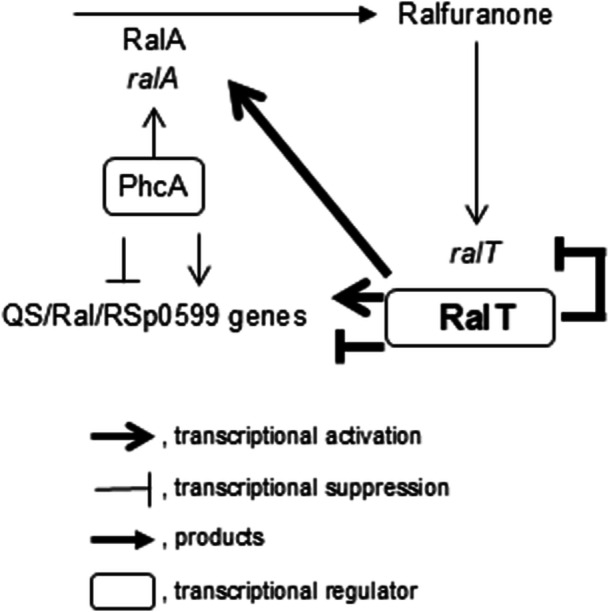

## 1 Introduction

Quorum sensing (QS) is a process whereby bacteria secrete and sense small molecules (i.e., QS signals) that accumulate in the extracellular space, regulating bacterial gene expression in response to fluctuations in cell population density (Ham 2013; Papenfort and Bassler 2016; Rutherford and Bassler 2012). Bacteria thus sense QS signal concentrations via specialized receptors, enabling them to estimate bacterial concentrations. Upon reaching a threshold concentration, bacteria activate QS to synchronously control the expression of genes that facilitate their adaptation to environmental conditions as well as virulence (Moreno‐Gámez et al. 2023).

The soil‐borne Gram‐negative beta‐proteobacterium *Ralstonia solanacearum* species complex (RSSC; Remenant et al. 2010) is composed of four phylotypes (I–IV), with three distinct species: *Ralstonia pseudosolanacearum* (phylotypes I and III), *R. solanacearum* (phylotype II), and *Ralstonia syzygii* (phylotype IV) (Safni et al. 2014). Notably, RSSC causes a devastating bacterial wilt disease that decreases crop production worldwide (Mansfield et al. 2012). Each RSSC strain produces methyl 3‐hydroxymyristate (3‐OH MAME) or methyl 3‐hydroxypalmitate as a QS signal via a reaction catalyzed by the methyltransferase PhcB (Flavier et al. 1997; Kai 2023; Kai et al. 2015; Ujita et al. 2019).

A phylotype I strain of RSSC, *R. pseudosolanacearum* strain OE1‐1 first attaches to the surface of the epidermis in tomato seedling root elongation zones and then colonizes the cortex surface and activates QS (Inoue et al. 2023; Tsuzuki et al. 2023). During the QS‐activation state, RSSC strains produce plant cell wall‐degrading enzymes (PCWDEs), such as β‐1,4‐cellobiohydrolase (CbhA; Liu et al. 2005; Senuma et al. 2023), endoglucanase (Roberts et al. 1988), and pectin methylesterase (Tans‐Kersten et al. 1998), and secreted PCWDEs via the type II secretion machinery (Tsujimoto et al. 2008). Strain OE1‐1 degrades the cell wall of cortical cells adjacent to the epidermis using PCWDEs to infect the damaged cortical cells and forms mushroom‐shaped biofilms, leading to the subsequent infection of xylem vessels and virulence (Inoue et al. 2023; Tsuzuki et al. 2023). QS‐deficient mutants did not infect the cortical cells and lost their virulence (Inoue et al. 2023; Senuma et al. 2023; Terazawa et al. 2024; Takemura et al. 2023; Tsuzuki et al. 2023). Therefore, QS is required for the virulence of strain OE1‐1 during an infection of tomato roots.

Strain OE1‐1 produces 3‐OH MAME as a QS signal (Kai 2023; Kai et al. 2015; Ujita et al. 2019). It is thought that the sensor histidine kinase PhcS assisted by the histidine kinase VsrA contributes to sensing 3‐OH MAME, leading to the autophosphorylation of the histidine at amino acid position 230 of PhcS (H230‐PhcS; Senuma et al. 2024). The putative cognate response regulators to PhcS, PhcR, PhcQ, and ChpA are required for the gene regulation by the LysR family transcriptional regulator PhcA (Takemura et al. 2021, 2023). Furthermore, though the phosphorylated H230‐PhcS is required for the full expression of *phcA* along with the alternative sensor histidine kinase PhcK, independently of 3‐OH MAME sensing (Senuma et al. 2020, 2025).

In the QS‐active strain OE1‐1, the activated PhcA controls the expression of genes (hereafter referred to as QS‐dependent genes) responsible for QS‐dependent activities, including virulence (Hikichi et al. 2017). Especially, activated PhcA induces expression of the major exopolysaccharide EPS I production‐related genes such as *xpsR* and *eps* operon (Garg et al. 2000), *lecM* encoding a lectin LecM (Mori et al. 2016), and *cbhA* encoding a PCWDE, CbhA (Senuma et al. 2023). During the QS‐active state, strain OE1‐1 thus induces the production of virulence‐related EPS I, LecM, and CbhA (Tsuzuki et al. 2023). EPS I is involved in the regulatory effects of PhcA on expression of QS‐dependent genes (Hayashi et al. 2019b). LecM is speculated to locate in outer membranes (Carter et al. 2024; Meng et al. 2015) and exhibits mannose, fructose, fucose, galactose, and arabinose affinities (Sudakevitz et al. 2002, 2004). Furthermore. LecM influences the volume of extracellularly secreted 3‐OH MAME (Hayashi et al. 2019a) as well as the attachment to plant surfaces (Mori et al. 2016). CbhA contributes to the full *phcA* expression (Senuma et al. 2023) as well as the invasion of cortical cells by strain OE1‐1. It is thus thought that these virulence‐related compounds participate in the feedback regulation of QS, contributing to virulence of strain OE1‐1 (Tsuzuki et al. 2023).

RSSC synthesizes aryl‐furanone secondary metabolites known as ralfuranones A, B, I, J, K, and L, which are secreted extracellularly (Kai 2023; Kai et al. 2014, 2016; Pauly et al. 2013). Ralfuranone I is a precursor for other ralfuranones; ralfuranones A, B, I, J, K, and L are hereafter referred to as ralfuranone. The expression of *ralA*, which encodes a ralfuranone synthase involved in the biosynthesis of ralfuranone, is induced by activated PhcA (Kai et al. 2014, 2016; Schneider et al. 2009; Wackler et al. 2011). During the QS‐active state, strain OE1‐1 thus induces the production of ralfuranone, which contributes to the development of mushroom‐shaped biofilms (Mori et al. 2018a). In addition, ralfuranone productivity‐deficiency leads to a change in the regulation of more than 80% of QS‐dependent genes, independently of QS, suggesting that the ralfuranone‐mediated regulation modulates the regulatory effects of PhcA on the expression of these QS‐dependent genes (Hayashi et al. 2019b; Mori et al. 2018b). The evidence indicates that ralfuranone is also involved in the feedback regulation of QS (Mori et al. 2018b; Tsuzuki et al. 2023).

To elucidate the mechanisms underlying the ralfuranone‐mediated gene regulation in strain OE1‐1, we first analyze the transcriptomes of the ralfuranone‐deficient *ralA*‐deletion (Δ*ralA*) and *phcA*‐deletion (Δ*phcA*) mutants conducting RNA‐sequencing (RNA‐seq) experiments. The generated data revealed that the expression of *RSp0599* (hereafter designated as *ralT*) encoding a novel type I TetR family (TetR/AcrR family) transcriptional regulator RalT decreased significantly in Δ*ralA*, but not in Δ*phcA*. We then generated a *ralT*‐deletion (Δ*ralT*) mutant and analyzed the transcriptomes of *R. pseudosolanacearum* strains by RNA‐seq. Furthermore, we examined their virulence as well as QS‐dependent phenotypes.

## 2 Materials and Methods

### 2.1 Bacterial Strains and Growth Conditions

We used *R. pseudosolanacearum* strain OE1‐1 (Table 1; Kanda et al. 2003) as well as the Δ*phcA* (Table 1; Mori et al. 2016) and Δ*ralA* (Kai et al. 2014) mutants of strain OE1‐1. All *R. pseudosolanacearum* strains were routinely grown in quarter‐strength M63 medium [4 mM (NH_4_)_2_SO_4_, 20 mM KH_2_PO_4_, 450 nM FeSO_4_·7H_2_O, 0.67 mM MgSO_4_, and 20 mM C_5_H_8_NO_4_Na·H_2_O] at 30°C. *Escherichia coli* strain DH5α (Table 1) and its transformants carrying recombinant plasmids (Table 1) were grown in Luria–Bertani medium (Hanahan 1983) at 37°C. The following antibiotics were used in selective media in the amounts indicated (μg/mL): gentamycin, 50; kanamycin, 50.

**Table 1:** Strains and plasmids used in this study.

	Relevant characteristics	Source
Plasmids		
pUC118	Amp^r^	Takara Bio
pHM1	Sp^r^, *oriV*	Innes et al. (1988)
pHM1‐GUS	pHM1 derivative carrying a gus gene	Ikawa and Tsuge (2016)
pralTproGUS	pHM1 derivative carrying 2.7‐kbp DNA fragment including the *ralT* promoter region‐fused *G*US	This study
pRSp0600proGUS	pHM1 derivative carrying 2.1‐kbp DNA fragment including the *RSp0600* promoter region‐fused *G*US	This study
pK18mobsacB	Km^r^, *oriT* (RP4), *sacB*, *lacZα*	Kvitko and Collmer (2011)
pdeltaralT	pK18mobsacB derivative carrying 1.6‐kbp DNA fragment for *RSp0599* deletion, Km^r^	This study
pUC18‐mini‐Tn*7*T‐Gm	Gm^r^	Choi et al. (2005)
pTNS2	helper plasmid carrying T7 transposase gene	Choi et al. (2005)
pralT	pUC18‐mini‐Tn*7*T‐Gm derivative carrying a 1.2‐kbp fragment including the promoter region and open reading frame of *ralT*	This study
pRSp0203proralT	pUC18‐mini‐Tn*7*T‐Gm derivative carrying a 1.4‐kbp fragment including the *RSp0203*promoter‐fused open reading frame of *ralT*	This study
pdelta‐xpsR	pK18mobsacB derivative carrying 1.3‐kbp DNA fragment for *xpsR* deletion, Km^r^	This study
pRSc0900proxpsR	pUC18‐mini‐Tn*7*T‐Gm derivative carrying a 2.1‐kbp fragment including the *RSc0900*promoter‐fused open reading frame of *xpsR*	This study
*Escherichia coli*		
DH5α	*recA1 endA1 gyrA96 thi‐1 hsdR17supE44* Δ(*lac*)*U169*(ϕ*80lac*ΔM15)	Takara Bio
*R. solanacearum*		
OE1‐1	Wild‐type strain, phylotype I, race 1, biovar 4,	Kanda et al. (2003)
Δ*phcA*	*phcA*‐deletion mutant of OE1‐1	Mori et al. (2016)
Δ*ralA*	*ralA*‐deletion mutant of OE1‐1	Kai et al. (2014)
Δ*ralT*	RSp0599‐deletion mutant of OE1‐1	This study
comp‐ralT	A transformant of Δ*ralT* with pralT, Gm^r^	This study
Δ*ralT* (*RSp0203pro::ralT*)	A transformant of Δ*ralT* with pRSp0203proralT, Gm^r^	This study
OE1‐1 (*ralTpro::GUS*)	A transformant of OE1‐1 with pralTproGUS, Sp^r^	This study
Δ*ralT* (*ralTpro::GUS*)	A transformant of Δ*ralT* with pralTproGUS, Sp^r^	This study
OE1‐1 (*RSp0600pro::GUS*)	A transformant of OE1‐1 with pRSp0600proGUS, Sp^r^	This study
Δ*ralT* (*RSp0600pro::GUS*)	A transformant of Δ*ralT* with pRSp0600proGUS, Sp^r^	This study
Δ*xpsR*	*xpsR*‐deletion mutant of OE1‐1	This study
Δ*phcA*/*xpsR*	*xpsR/phcA*‐deletion mutant of OE1‐1	This study
Δ*phcA* (*xpsR*)	A transformant of Δ*phcA* with pRSc0900proxpsR, Gm^r^	This study
Δ*xpsR* (*xpsR*)	A transformant of Δ*xpsR* with pRSc0900proxpsR, Gm^r^	This study
Δ*phcA*/*xpsR* (*xpsR*)	A transformant of Δ*phcA*/*xpsR* with pRSc0900proxpsR, Gm^r^	This study
Δ*ralT* (*xpsR*)	A transformant of Δ*ralT* with pRSc0900proxpsR, Gm^r^	This study

Abbreviations: Gm^r^, gentamycin resistant; Km^r^, kanamycin resistant; Sp^r^, spectinomycin resistant.

### 2.2 General DNA Manipulations

*Ralstonia pseudosolanacearum* was transformed by electroporation as described previously (Allen 1991). Double‐stranded DNA sequencing templates were pre‐pared with Fast GeneTM Plasmid miniprep kits (NIPPON Genetics, Tokyo, Japan) and sequences were determined using an Automated DNA Sequencer Model 373 (Applied Biosystems, Foster City, CA, USA). DNA sequence data were analyzed using the DNASYS‐Mac software (Hitachi Software Engineering, Yokohama, Japan). Enzymes, including restriction endonucleases (Takara, Ohtsu, Japan), were used according to the manufacturer's instructions.

### 2.3 Generation of Δ*ralT* and Δ*ralT* Transformants

To generate the Δ*ralT* mutant (Table 1), we created a plasmid, pdeltaralT (Table 1), by inserting a recombinant fragment derived from a PCR amplification using specific primers (Table 2) into pK18mobsacB (Kvitko and Collmer 2011). Plasmid pdeltaralT was electroporated into strain OE1‐1 competent cells, which were prepared as previously described by Allen (1991). A kanamycin‐sensitive, sucrose‐resistant recombinant, Δ*ralT*, was then selected.

**Table 2:** Primers used for generation of plasmids in this study.

Plasmid	Primers	Nucleotide sequences
pdeltaralT	RSp0599‐1FW	5ʹ‐CGgaattcTTGTAGCGCATCAGCCTGTC‐3ʹ
	RSp0599‐1RV	5ʹ‐GATCCAGCTATAGCATGTCATCTCCACGTGGACAC‐3ʹ
	RSp0599‐2FW	5ʹ‐GGAGATGACATGCTATAGCTGGATCGCCGGC‐3ʹ
	RSp0599‐2RV	5ʹ‐CGggatccGCTTTGCGGTGGAAGAGGTG‐3ʹ
pralT	RSp0599pro‐FW	5ʹ‐GTtctagaGCTCGGGAACCGGCATCGGCA‐3ʹ
	RSp0599pro‐RV	5ʹ‐GTTgagctcCTATAGGCCGTTCTCCACCA‐3ʹ
pRSp0203proralT	RSp0203‐1FW	5ʹ‐GTTctgcagGAGAACGGGCATGAGTGACC‐3ʹ
	RSp0203‐1RV	5ʹ‐GCTGGGTCATTCTGCCTGAAAGCTGCTGCG‐3ʹ
	RSp0203‐2FW	5ʹ‐TTCAGGCAGAATGACCCAGCAGCACGCCC‐3ʹ
	RSp0203‐2RV	5ʹ‐GTTgagctcCTATAGGCCGTTCTCCACCA‐3ʹ
pralTproGUS	RSp0599pro‐FW	5ʹ‐GTTctgcagGGCGCCGGCATGCTCGG‐3ʹ
	RSp0599pro‐RV	5ʹ‐GTTggtaccCAGGGCAATGCCCGGCATC‐3ʹ
pRSp0600proGUS	RSp0600pro‐FW	5ʹ‐GTTctgcag*G*GTCATCTCCACGTGGACACG‐3ʹ
	RSp0600pro‐RV	5ʹ‐GTTggtacc*C*ACATCCTTGGCGCGGAC‐3ʹ
pdelta‐xpsR	xpsR‐1FW	5ʹ‐CGCggtaccTGCCGAATCAATCAAACCGAGAG‐3ʹ
	xpsR‐1RV	5ʹ‐CTTGCTGAGTCACATTTCGGTAATTTCCCTCCGG‐3ʹ
	xpsR‐2FW	5ʹ‐GAATTACCGAAATGTGACTCAGCAAGATGCCGG‐3ʹ
	xpsR‐2RV	5ʹ‐CCCaagcttGCATGACCACGGCGTTC‐3ʹ
pRSc0900proxpsR	pUCxpsR ‐1FW	5ʹ‐GGggtaccAGATGCCGTAGATGATGCC‐3ʹ
	pUCxpsR ‐1RV	5ʹ‐CTTCTGTTCCATATTTCTCCTCTCAGGATGAG‐3ʹ
	pUCxpsR ‐2FW	5ʹ‐GAGAGGAGAAATATGGAACAGAAGCTCATCTTCTC‐3ʹ
	pUCxpsR ‐2RV	5ʹ‐CCCaagcttCGGTTTAGTTGA ATGTGGCTG‐3ʹ

*Note:* lowercase, restriction enzyme sites.

To generate the comp‐ralT transformant containing the native *ralT* promoter region and open reading frame, we created a plasmid, pralT (Table 1), by inserting a recombinant fragment obtained by PCR using specific primers (Table 2) into pUC18‐mini‐Tn*7*T‐Gm (Table 1; Choi et al. 2005). This plasmid was electroporated into Δ*ralT* cells along with the T7 transposase expression vector pTNS2 (Choi et al. 2005), after which a gentamycin‐resistant transformant, comp‐ralT, was selected.

To generate plasmid pRSp0203proralT, which includes the *RSp0203* promoter fused to the *ralT* open reading frame, we completed a PCR amplification of a recombinant fragment using specific primers (Table 2) and ligated it into pUC18‐mini‐Tn*7*T‐Gm. We then electroporated pRSp0203proralT into Δ*ralT* cells along with pTNS2, after which a gentamycin‐resistant transformant, Δ*ralT* (*RSp0203pro::ralT*) was selected.

### 2.4 Assay of *ralT* and *RSp0600* Promoter Activities

To analyze *ralT* and *RSp0600* promoter activities, we first amplified DNA fragments comprising the *ralT* and *RSp0600* promoter regions by PCR using specific primers (Table 2). The amplified fragments were digested with PstI (Takara, Ohtsu, Japan) and KpnI (Takara) and ligated into PstI‐ and KpnI‐digested pHM1‐GUS (Ikawa and Tsuge 2016) derived from pHM1 (Innes et al. 1988) to generate plasmids pralTproGUS and pRSp0600proGUS. The recombinant plasmids were electroporated into strain OE1‐1 and Δ*ralT* competent cells. Spectinomycin‐resistant OE1‐1 (*ralTpro::GUS*) and Δ*ralT* (*ralTpro::GUS*) as well as OE1‐1 (*RSp0600pro::GUS*) and Δ*ralT* (*RSp0600pro::GUS*) were then selected.

We measured the GUS activity of *R. pseudosolanacearum* strains according to a modified version of the method described by Tsuge et al. (2002). Briefly, bacterial strains were grown in quarter‐strength M63 medium until the OD_600_ reached 1.0, after which 75 μL culture was added to an equal volume of 2 × assay buffer (0.1 M phosphate buffer pH 7.0, 0.15% *β*‐mercaptoethanol, and 0.2% Triton X‐100) containing 2 mM *p*‐nitrophenyl *β*‐d‐glucuronide as the substrate. After an incubation at 37°C for 2 h, the culture was centrifuged at 15,000 rpm. Pelleted bacterial cells were removed and the absorbance of the supernatant was measured at 415 nm (A_415_). The analysis was completed using eight technical replicates. Means were analyzed for significant differences between *R. pseudosolanacearum* strains via an analysis of variance (ANOVA) based on Tukey–Kramer's honestly significant difference test (*p* < 0.05).

### 2.5 Generation of the Δ*xpsR* Mutant and Its Transformants With Ectopic Expression of *xpsR*

To create *xpsR*‐deletion mutants, we first generated the pK18mobsacB‐based plasmid pdelta‐xpsR (Table 1) by inserting a recombinant fragment derived from a PCR amplification using specific primers (Table 2) into pK18mobsacB. Next, pdelta‐xpsR was electroporated into strain OE1‐1 and Δ*phcA* competent cells and then the kanamycin‐sensitive, sucrose‐resistant recombinants *xpsR*‐deletion (Δ*xpsR*), and *xpsR* and *phcA*‐deletion (Δ*phcA*/*xpsR*) mutants were selected.

To create transformants with ectopic expression of *xpsR*, we generated the pUC18‐mini‐Tn*7*T‐GM‐based plasmid pRSc0900proxpsR (Table 1) carrying the *RSc0900* promoter fused to *xpsR* by inserting a recombinant fragment obtained from a PCR amplification using specific primers (Table 2) into pUC18‐mini‐Tn*7*T. Plasmid pRSc0900proxpsR was electroporated into Δ*phcA*, Δ*xpsR*, Δ*phcA*/*xpsR*, and Δ*ralT* competent cells to produce the spectinomycin‐resistant transformants Δ*phcA* (*xpsR*), Δ*xpsR* (*xpsR*), Δ*phcA*/*xpsR* (*xpsR*), and Δ*ralT* (*xpsR*), respectively.

### 2.6 qRT‐PCR

Total RNA was extracted from *R. pseudosolanacearum* strains that were grown in quarter‐strength M63 medium until the OD_600_ reached 0.3 using a High Pure RNA Isolation kit (Roche Diagnostics, Tokyo, Japan) and DNase I (Takara) at concentration of 167 nU/μL as previously described (Hayashi et al. 2019a). A quantitative real‐time polymerase chain reaction (qRT‐PCR) analysis involving gene‐specific primers (Table 3) was completed using a SYBR GreenER qPCR Reagent system (Invitrogen, Tokyo, Japan) and a 7300 Real‐Time PCR platform (Applied Biosystems, Foster City, CA, USA). The *rpoD* transcript level was used as the internal standard to normalize all values and 2‐ΔΔCt for the calculation of gene expression. There were no significant differences in the *rpoD* transcript level among *R. pseudosolanacearum* strains. Means were analyzed for significant differences between *R. pseudosolanacearum* strains by an ANOVA based on Tukey–Kramer's honestly significant difference test (*α* = 0.05).

**Table 3:** Primers used in the quantitative real‐time polymerase chain reaction assays.

Genes	Primers	Nucleotide sequences
*rpoD*	rpoD‐FW	5ʹ‐ATCGTCGAGCGCAACATCCC‐3ʹ
	rpoD‐RV	5ʹ‐AGATGGGAGTCGTCGTCGTCGTG‐3ʹ
*fliC*	fliC‐RV2	5ʹ‐ATTGGAAGGTCGTCGAAGCCAC‐3ʹ
	fliC‐FW2	5ʹ‐CAAACGCAAGGTATTCAGAACG‐3ʹ
*ralT*	RSp0599‐qPCR‐FW	5ʹ‐TTTGCGCAACAGGAGACC‐3ʹ
	RSp0599‐qPCR‐RV	5ʹ‐AGCAACTGGAACGGATTGAG‐3ʹ
*RSp0600*	RSp0600‐RT‐FW43	5ʹ‐CGTGCGCTGATCCAGGAATG‐3ʹ
	RSp0600‐RT‐RV209	5ʹ‐CGAACCAGTCCTGCCAGTTG‐3ʹ
*xpsR*	xpsR‐qPCR‐FW	5ʹ‐TCTTCTCGCGCGAACA‐3ʹ
	xpsR‐qPCR‐RV	5ʹ‐AACCAGCGACTCTGTC‐3ʹ
*epsB*	epsB‐FW	5ʹ‐ATGGTCGAGCTGATGGATA‐3ʹ
	epsB‐RV2	5ʹ‐TGGAGCTGCTTGATCGTCTC‐3ʹ

### 2.7 Transcriptome Analysis Based on RNA‐Seq

We eliminated ribosomal RNA from the extracted total RNA of *R. pseudosolanacearum* strains using a Ribo‐Zero rRNA Removal kit (Gram‐negative bacteria) (Illumina, Madison, WI, USA). An oriented, paired‐end RNA‐seq (2 × 100 bp) analysis was then performed using an Illumina HiSeq. 2500 system (Illumina) and a DNBSEQ‐G400 system (MGI Tech, Shenzhen, China) by Bioengineering Lab Co. (Sagamihara, Japan). The generated reads were trimmed using Cutadapt (Martin 2011) and Trimmomatic (Bolger et al. 2014) and then mapped to the GMI1000 strain genome (Salanoubat et al. 2002) using the TopHat program (Trapnell et al. 2009). We analyzed at least three independent biological replicates per strain.

### 2.8 GO Enrichment Analysis

RNA‐seq data were statistically analyzed in the R environment (R Core Team 2022). We excluded genes with zero counts in at least one strain OE1‐1 sample in the raw count data set. RNA‐seq read counts of the remaining genes were normalized using the function calcNormFactors (trimmed mean of *M*‐values normalization) in the package edgeR (Robinson et al. 2010). To extract genes with significant changes in transcript levels, the following thresholds were applied: *q*‐value < 0.05 and |log_2_(fold‐change)| ≥ 2. The false discovery rate (*q*‐value) was calculated from *p*‐values estimated by edgeR according to the Benjamini–Hochberg method (Benjamini and Hochberg 1995). Hierarchical clustering of all normalized mean transcript values based on their relative transcript levels (counts per million) was performed using Cluster3.0 software (de Hoon et al. 2004). The average of at least three replicates per strain was calculated. Heatmaps were created using TreeView (Eisen et al. 1998). GO terms were obtained from the QuickGO database (https://www.ebi.ac.uk/QuickGO/annotations) using *R. solanacearum* GMI1000 gene annotation data. A GO enrichment analysis was conducted using the R package goseq (Young et al. 2010). Fold enrichments were calculated as follows: the ratio of differentially expressed genes (DEGs) annotated with a specific GO term to the expected ratio for all genes. Scores were calculated as follows: the proportion of all DEGs annotated with a GO term was divided by the proportion of all genes annotated with that GO term.

### 2.9 Swimming Motility Assay

For the swimming motility assay, 5‐μl aliquots of *R*. *pseudosolanacearum* cell suspensions at a cell density of 5.0 × 10^5^ colony‐forming units (cfu)/mL were added to the surface of quarter‐strength M63 medium solidified with 0.25% w/v agar (Mori et al. 2018b). Swimming area diameters were measured after a 48 h incubation. Fifteen technical replicates were prepared per group. Means were analyzed for significant differences between *R. pseudosolanacearum* strains by an ANOVA based on Tukey–Kramer's honestly significant difference test (*α* = 0.05).

### 2.10 EPS I Production Assay

EPS I produced by *R. pseudosolanacearum* cells grown on quarter‐strength M63 medium solidified with 1.5% agar was analyzed in an enzyme‐linked immunosorbent assay (Agdia, Elkhart, IN, USA) and quantified according to A_650_ as previously described (Mori et al. 2016). Twenty‐one technical replicates were prepared per group. Means were analyzed for significant differences between *R. pseudosolanacearum* strains by an ANOVA based on Tukey–Kramer's honestly significant difference test (*α* = 0.05).

### 2.11 Virulence Assays

Tomato plants (*Solanum lycopersicum* cultivar Ohgata‐Fukuju; Marutane, Kyoto, Japan) were grown in pots containing a mixture of vermiculite and peat moss (3: 1) in a growth room at 25°C under 10,000 lx for 16 h per day; they were watered with fivefold‐diluted Hoagland's solution (Hikichi et al. 1999). Eighteen‐day‐old tomato plants were inoculated with *R. pseudosolanacearum* strains at 1.0 × 10^8^ cfu/ml using a root‐dip method as previously described (Mori et al. 2016). We monitored plants daily for wilting symptoms, which were rated according to the following disease index scale and plants with at least 50% of wilting were considered as dead for the statistical survival analysis: 0, no wilting; 1, 1%–25% wilting; 2, 26%–50% wilting; 3, 51%–75% wilting; 4, 76%–99% wilting; 5, dead. For each bacterial strain, 20 technical replicates were tested. Means were analyzed for significant differences between *R. pseudosolanacearum* strains by an ANOVA based on Tukey–Kramer's honestly significant difference test (*α* = 0.05). Disease scoring was transformed into binary data, with a disease index below 3 corresponding to 0 and a disease index equal to or higher than 3 corresponding to 1 (Perrier et al. 2019). Log‐rank pairwise tests were used to determine significant difference between strains. A *p*‐value smaller than 0.05 was considered significant, indicating that the Ho hypothesis of similarity of the survival experience of the tested strains can be rejected. Statistical analyses were done with RStudio (Posit team 2025).

## 3 Results

### 3.1 Identification of RalT as a Novel TetR/AcrR Family Transcriptional Regulator

**Figure 1:**
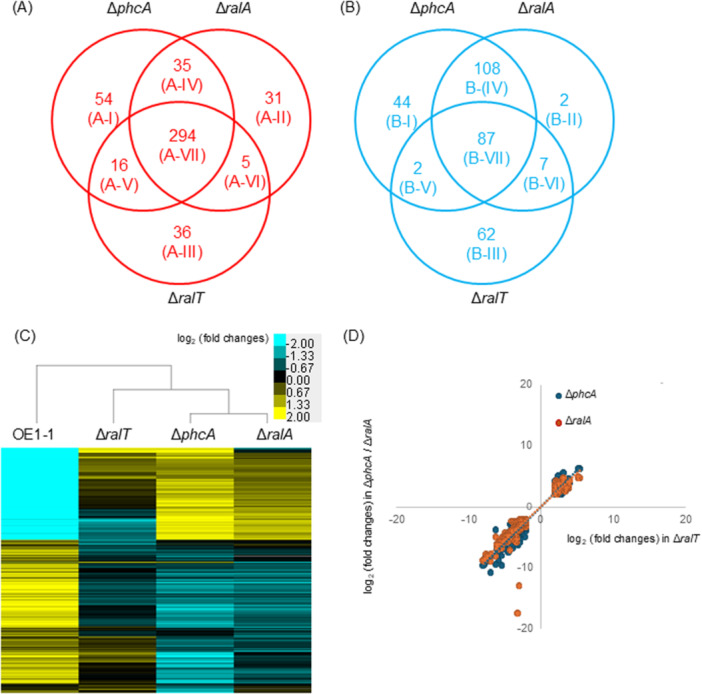
RNA‐sequencing analysis of the transcriptomes of *Ralstonia pseudosolanacearum* strain OE1‐1 and *phcA*‐deletion (Δ*phcA*), *ralA*‐deletion (Δ*ralA*), and *ralT*‐deletion (Δ*ralT*) mutants grown in quarter‐strength M63 medium until the OD600 reached 0.3. (A) Number of genes with transcript‐level log_2_(fold‐change) ≤ −2 in Δ*phcA*, Δ*ralA*, and Δ*ralT* (relative to their transcript levels in OE1‐1) (*q* < 0.05). Roman numerals in parentheses are responsive to clusters in Table 4. (B) Number of genes with transcript‐level log2(fold‐change) ≥ 2 in Δ*phcA*, Δ*ralA*, and Δ*ralT* (relative to their transcript levels in OE1‐1) (*q* < 0.05). Roman numerals in parentheses are responsive to clusters in Table 5. (C) Hierarchical clustering of the relative expression of QS/Ral/p0599‐dependent genes in *R*. *pseudosolanacearum* strains. (D) Correlations among the expression levels of QS/Ral/p0599‐dependent genes revealed by the comparison of Δ*ralT* with Δ*phcA* and Δ*ralA*.

We first performed an RNA‐seq analysis of the transcriptomes of the Δ*phcA* and Δ*ralA* mutants and strain OE1‐1 that were grown in quarter‐strength M63 medium until the optical density at 600 nm (OD_600_) reached 0.3. On the basis of the mapping of RNA‐seq reads for strain OE1‐1 to the GMI1000 genome (Salanoubat et al. 2002), we identified 4327 protein‐coding transcripts (Table S1). The following thresholds were applied to identify genes with transcript levels in the Δ*phcA* and Δ*ralA* mutants that differed significantly from those in strain OE1‐1: *q*‐value < 0.05 and |log_2_(fold‐change)| ≥ 2. The expression levels of 399 genes and 241 genes decreased and increased significantly, respectively, in the Δ*phcA* mutants (hereafter referred to as positively and negatively QS‐dependent genes) (Figure 1A,B; Table S2). Among the QS‐dependent genes, the expression levels of 329 genes (hereafter referred to as positively QS/Ral‐dependent genes) and 195 genes (hereafter referred to as negatively QS/Ral‐dependent genes) decreased and increased significantly, respectively, in the Δ*ralA* mutants (Figure 1A,B; Table S3). These results suggest that the regulation of 82.4% and 80.9% of positively and negatively QS‐dependent genes, respectively, are ralfuranone‐mediated, which is consistent with the findings of our previous study (Mori et al. 2018b), although the underlying mechanisms are unclear.

**Figure 2:**
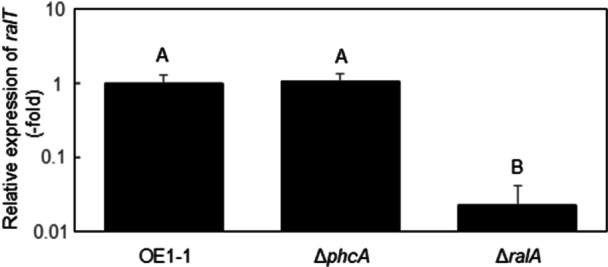
The *ralT* expression level in *Ralstonia pseudosolanacearum* strain OE1‐1, *phcA*‐deletion (Δ*phcA*), and *ralA*‐deletion (Δ*ralA*) mutants according to a quantitative real‐time polymerase chain reaction analysis. For assays of the *ralT* expression level, strains were grown in quarter‐strength M63 medium until the optical density at 600 nm (OD_600_) reached 0.3 and then used for an RNA extraction. The *rpoD* transcript level was used as the internal standard to normalize all values and 2‐ΔΔCt for the calculation of *ralT* expression level. There were no significant differences in the *rpoD* transcript level among *R. pseudosolanacearum* strains. Experiments were conducted using five biological replicates. Bars indicate standard errors. Means were analyzed for significant differences between strains via an ANOVA followed by Tukey–Kramer's honestly significant difference test. Statistically significant differences are indicated by different lowercase letters (*α* = 0.05).

**Figure 3:**
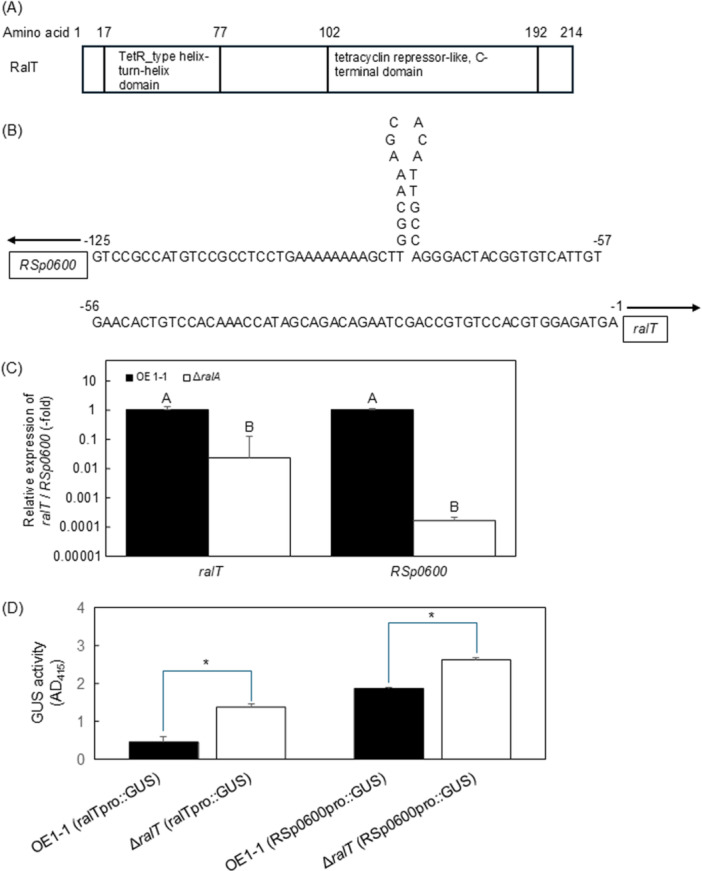
A novel type I TetR family (TetR/AcrR family) transcriptional regulator (RalT) of *Ralstonia pseudosolanacearum* strain OE1‐1. (A) Domains in RalT were identified by analyzing deduced amino acid sequences using a BLAST algorithm and InterProScan. (B) *ralT* and *RSp0600* promoter sequences. (C) The expression level of *ralT* and *RSp0600* in strain OE1‐1 and *ralA*‐deletion (Δ*ralA*) mutants according to a quantitative real‐time polymerase chain reaction analysis. For assays of the expression levels of *ralT* and *RSp0600*, strains were grown in quarter‐strength M63 medium until the optical density at 600 nm (OD_600_) reached 0.3 and then used for an RNA extraction. The *rpoD* transcript level was used as the internal standard to normalize all values and 2‐ΔΔCt for the calculation of *ralT* expression level. There were no significant differences in the *rpoD* transcript level among *R. pseudosolanacearum* strains. Experiments were conducted using five biological replicates. Bars indicate standard errors. Means were analyzed for significant differences between strains via an ANOVA followed by Tukey–Kramer's honestly significant difference test. Statistically significant differences are indicated by different lowercase letters (*α* = 0.05). (D) Effect of RalT on *ralT* and *RSp0600* promoter activities. We measured the absorbance at 415 nm (A_415_) to determine the GUS activity of the transformants [OE1‐1 (*ralTpro::GUS*), Δ*ralT* (*ralTpro::GUS*)] of strain OE1‐1 and the *ralT*‐deletion (Δ*ralT*) mutant, with the *ralT* promoter fused to *GUS*, and the transformants [OE1‐1 (*RSp0600pro::GUS*), Δ*ralT* (*RSp0600pro::GUS*)] of strain OE1‐1 and Δ*ralT*, with the *RSp0600* promoter fused to *GUS*. Eight technical replicates were prepared. Means were analyzed for significant differences between *R. pseudosolanacearum* strains via an ANOVA followed by Tukey–Kramer's honestly significant difference test (*α* = 0.05).

To identify the transcriptional regulator‐encoding genes involved in the ralfuranone‐mediated regulation of QS‐dependent genes, we identified the 36 genes with expression levels that decreased significantly in Δ*ralA*, but not in Δ*phcA*, included a putative transcriptional regulator‐encoding gene (*ralT*) (Figure 1A; Table S4). A quantitative real‐time polymerase chain reaction (qRT‐PCR) assay also showed that the expression level of *ralT* in Δ*ralA*, but not in Δ*phcA*, significantly reduced, compared to that in strain OE1‐1 (*α* = 0.05; Figure 2). An analysis of the RalT amino acid sequence deduced according to the strain OE1‐1 genome using the BLAST algorithm and InterProScan indicated that RalT has a highly conserved N‐terminal helix‐turn‐helix motif and a C‐terminal tetracycline repressor‐like domain (Figure 3A), similar to type I TetR family (TetR/AcrR family) transcriptional regulators (Ahn et al. 2012; Colclough et al. 2019; Cuthbertson and Nodwell 2013; Ramos et al. 2005). Many TetR/AcrR family transcriptional regulators act as repressors by binding palindromic sequences overlapping promoters, thereby preventing the recruitment and binding of RNA polymerase. We detected a palindromic sequence (5′‐TGGCAAAGCACATTGCCA‐3′; Figure 3B) in the putative *ralT* promoter region. Furthermore, a qRT‐PCR analysis showed that the expression levels of *ralT* and *RSp0600* in Δ*ralA* significantly reduced, compared to those in OE1‐1 (*α* = 0.05; Figure 3C). We thus hypothesized that RalT may bind and repress the expression of both *ralT* and *RSp0600* (genes with divergent promoters). To test this hypothesis, we used pHM1 (Gu et al. 2005) to generate recombinant plasmids pralTproGUS and pRSp0600proGUS, which contained a β‐glucuronidase (GUS)‐encoding gene under the control of the *ralT* promoter (*ralTpro::GUS*) and the *RSp0600* promoter (*RSp0600pro::GUS*), respectively. We then transformed strain OE1‐1 and the Δ*ralT* mutant with these recombinant plasmids. Transformants were selected according to spectinomycin resistance and analyzed in terms of GUS activity. The deletion of *ralT* significantly enhanced GUS activity derived from both *ralTpro::GUS* and *RSp0600pro::GUS* (Means were analyzed for significant differences between *R. pseudosolanacearum* strains via an ANOVA based on Tukey–Kramer's honestly significant difference test (*α* = 0.05; Figure 3D), suggesting that RalT is a repressor that negatively regulates *RSp0600* and *ralT* expression.

### 3.2 RNA‐Seq Analysis of the Δ*ralT* Transcriptome

To characterize RalT as a transcriptional regulator, we conducted an RNA‐seq analysis of the Δ*ralT* transcriptome. A comparison with strain OE1‐1 revealed a lack of significant changes in the expression levels of QS‐related genes, such as *phcB*, *phcK*, *phcS*, *vsrA*, *phcQ*, *phcR*, and *chpA*, in the Δ*ralT* mutant (Table S5). However, in the Δ*ralT* mutant, 351 genes (hereafter referred to as positively p0599‐dependent genes) had significantly downregulated expression levels, whereas 158 genes (hereafter referred to as negatively p0599‐dependent genes) had significantly upregulated expression levels (Figure 1A,B; Table S6). Of the positively and negatively p0599‐dependent genes, 310 and 89 genes, respectively, were included among the positively and negatively QS‐dependent genes (hereafter referred to as positively and negatively QS/p0599‐dependent genes). Furthermore, of the positively and negatively QS/p0599‐dependent genes, expression level of 294 and 87 genes, respectively, decreased and increased significantly, after the *ralA*‐deletion (hereafter referred to as positively and negatively QS/Ral/p0599‐dependent genes) (Figure 1A,B; Table S7).

A dendrogram for the hierarchical clustering of *R. pseudosolanacearum* strains was created on the basis of relative expression levels normalized against the expression of QS/Ral/p0599‐depenent genes, which revealed that the Δ*ralT* mutant was clustered with the Δ*phcA* and Δ*ralA* mutants (Figure 1C). Furthermore, QS/Ral/p0599‐dependent gene transcript levels were positively correlated between Δ*ralA* and Δ*ralT* [y, log_2_(fold‐change) in Δ*ralA*; x, log_2_(fold‐change) in Δ*ralT*; *y* = 1.1056x + 0.0322, *r* ^2^ = 0.9024, Figure 1D]. Additionally, QS/Ral/p0599‐dependent gene transcript levels were highly positively correlated between Δ*phcA* and Δ*ralT* [y, log_2_(fold‐change) in Δ*phcA*; x, log_2_(fold‐change) in Δ*ralT*; *y* = 1.201x − 0.0137, *r* ^2^ = 0.9457].

Data in the transcriptome analysis of Δ*ralT* as well as Δ*phcA* and Δ*ralA* suggest that RalT may participate in the regulation of 89.4% of positively QS/Ral‐dependent genes and 44.6% of negatively QS/Ral‐dependent genes (Figure S1).

### 3.3 Gene Ontology Enrichment Analysis of QS/Ral/p0599‐Dependent Genes

To characterize the functions of proteins encoded by QS/Ral/p0599‐dependent genes, we performed a Gene Ontology (GO) enrichment analysis of the QS/Ral/p0599‐dependent genes (cluster A‐VII; Figure 1A). For the positively QS/Ral/p0599‐dependent genes, typical GO terms, including “lipopolysaccharide biosynthetic process” were enriched among 163 genes affected by the genotype (Table 4). In the GO term, “quorum sensing,” which are the SolI/R and RasI/R systems using *N*‐acyl‐homoserine lactones (ATL) as QS signals (Li et al. 2024), is included, suggesting that the QS using 3‐OH MAME as a QS signal regulates the AHL‐based QS systems.

**Table 4:** The Gene Ontology (GO) terms enriched in genes which were downregulated in *Ralstonia pseudosolanacearum phcA*‐deletion (Δ*phcA*), *ralA*‐deletion (Δ*ralA*), and *ralT*‐deletion (Δ*ralT*) mutants, compared to strain OE1‐1.

Cluster^a^	GO term	*p* value	Fold enrichment	numDEInCat
A‐II	phenylacetate catabolic process	2.82E‐06	59.8202765	3
	maltose alpha‐D‐glucosyltransferase activity	0.00287586	139.5806452	1
	phenylacetyl‐CoA 1,2‐epoxidase activity	0.0038603	139.5806452	1
A‐III	histidine catabolic process to glutamate and formamide	0.00031422	60.09722222	2
	histidine catabolic process to glutamate and formate	0.00031422	60.09722222	2
	propionate catabolic process, 2‐methylcitrate cycle	0.00052095	48.07777778	2
	allantoin metabolic process	0.00621749	120.1944444	1
	urate catabolic process	0.00621749	120.1944444	1
	2‐oxo‐4‐hydroxy‐4‐carboxy‐5‐ureidoimidazoline decarboxylase activity	0.00621749	120.1944444	1
	ribokinase activity	0.00681115	120.1944444	1
	D‐ribose catabolic process	0.00681115	120.1944444	1
	1‐phosphofructokinase activity	0.00681533	120.1944444	1
	formimidoylglutamase activity	0.00685296	120.1944444	1
	xylan catabolic process	0.0068655	120.1944444	1
	metal ion binding	0.00690893	2.907930108	6
	ethylene biosynthetic process	0.00693655	120.1944444	1
	2‐oxoglutarate oxygenase/decarboxylase (ethylene‐forming) activity	0.00693655	120.1944444	1
	fumarylacetoacetase activity	0.00726208	120.1944444	1
	acetaldehyde dehydrogenase (acetylating) activity	0.00760752	120.1944444	1
	catalase activity	0.00761999	120.1944444	1
	hydrogen peroxide catabolic process	0.00761999	120.1944444	1
	urocanate hydratase activity	0.00800165	120.1944444	1
A‐IV	D‐xylose transmembrane transport	0.00496513	123.6285714	1
	monosaccharide binding	0.00496513	123.6285714	1
	4‐hydroxybutyrate dehydrogenase activity	0.00990697	61.81428571	1
	mannose‐6‐phosphate isomerase activity	0.00990827	61.81428571	1
A‐VI	citrate synthase activity	0.00145618	865.4	1
	propionate metabolic process, methylcitrate cycle	0.00486466	432.7	1
	ATPase‐coupled cation transmembrane transporter activity	0.00840014	288.4666667	1
A‐VII	lipopolysaccharide biosynthetic process	0.00012595	9.811791383	4
	integral component of membrane	0.00033515	1.130009682	82
	oxidoreductase activity	0.00153168	1.925678683	14
	acylphosphatase activity	0.00259002	14.71768707	2
	polysaccharide transport	0.00317369	14.71768707	2
	cellulose catabolic process	0.00370254	14.71768707	2
	tRNA aminoacylation for protein translation	0.00374334	14.71768707	2
	extracellular space	0.00409855	14.71768707	2
	heme binding	0.00633789	2.354829932	8
	quorum sensing	0.00750931	9.811791383	2
A‐VII	organonitrogen compound biosynthetic process	0.0079367	9.811791383	4

^a^ Clusters are referred to parentheses in Figure 1A.

For the negatively QS/Ral/p0599‐dependent genes (cluster B‐VII), GO terms related to bacterial motility, including “chemotaxis,” “bacterial‐type flagellum basal body,” and “bacterial‐type flagellum‐dependent cell motility,” were enriched among 312 genes affected by the genotype (Figure 1B). Other terms, such as “siderophore biosynthetic process” and “siderophore uptake transmembrane transporter activity” were enriched (Table 5). The GO term “viral capsid assembly” was included in the cluster B‐III, of which genes were included in the negatively p0599‐dependent genes but not the negatively QS‐dependent genes and Ral‐dependent genes.

**Table 5:** The Gene Ontology (GO) terms enriched in genes which were upregulated in *Ralstonia pseudosolanacearum phcA*‐deletion (Δ*phcA*), *ralA*‐deletion (Δ*ralA*), and *ralT*‐deletion (Δ*ralT*) mutants, compared to strain OE1‐1.

Cluster^a^	GO term	*p* value	Fold enrichment	numDEInCat
B‐I	structural constituent of ribosome	0	23.24421488	13
	translation	0	20.29256854	13
	ribosome	2.71E‐11	21.07305195	9
	rRNA binding	3.18E‐05	12.60780886	5
	large ribosomal subunit rRNA binding	7.32E‐05	98.34090909	2
	succinate‐CoA ligase (ADP‐forming) activity	7.82E‐05	98.34090909	2
	regulation of translation	0.00043465	49.17045455	2
	tRNA binding	0.00054648	13.56426332	4
	small ribosomal subunit	0.00107487	32.78030303	2
	nitrate metabolic process	0.00109517	98.34090909	2
	nitrate reductase complex	0.00184183	65.56060606	2
	large ribosomal subunit	0.0019857	24.58522727	2
	transmembrane signaling receptor activity	0.00205597	15.52751196	3
	translation elongation factor activity	0.00272986	32.78030303	2
	RNA helicase activity	0.0045057	39.33636364	2
	nitrate reductase activity	0.00535516	39.33636364	2
	ATP‐dependent activity, acting on DNA	0.00558897	28.0974026	2
	signal transduction	0.00702577	11.34702797	3
	methylenetetrahydrofolate reductase NADH activity	0.00869301	98.34090909	1
	methylenetetrahydrofolate reductase NADPH activity	0.00869301	98.34090909	1
	NADH dehydrogenase activity	0.00984111	98.34090909	1
	NADH oxidation	0.00984111	98.34090909	1
B‐III	viral capsid assembly	1.05E‐06	69.79032258	3
	inorganic phosphate transmembrane transporter activity	1.44E‐06	52.34274194	3
	phosphate ion transmembrane transport	0.00014358	46.52688172	2
	site‐specific DNA‐methyltransferase (cytosine‐N4‐specific) activity	0.00686045	69.79032258	1
	ATPase‐coupled phosphate ion transmembrane transporter activity	0.00807828	69.79032258	1
B‐IV	structural constituent of ribosome	0	10.92676768	15
	translation	0	9.539241623	15
	rRNA binding	0	12.32763533	12
	ribosome	3.73E‐09	8.58531746	9
	protein secretion by the type III secretion system	1.10E‐08	33.38734568	5
	modulation by symbiont of host defense‐related programmed cell death	1.93E‐08	33.38734568	5
	large ribosomal subunit	2.21E‐06	20.03240741	4
	signal transduction	0.00044029	7.70477208	5
	extracellular region	0.00053432	10.0162037	4
	protein secretion	0.00103767	7.284511785	4
	transmembrane signaling receptor activity	0.00127946	8.434697856	4
	type III protein secretion system complex	0.00149349	26.70987654	2
	small ribosomal subunit	0.00271095	13.35493827	2
	cytochrome bo3 ubiquinol oxidase activity	0.00281254	13.35493827	2
	chemotaxis	0.006475	4.85634119	4
	protein targeting	0.00984047	10.0162037	2
B‐V	chemotaxis	0.00878679	65.56060606	1
B‐VI	siderophore uptake transmembrane transporter activity	0.00577146	88.30612245	1
	signaling receptor activity	0.00577146	88.30612245	1
B‐VII	cytoskeletal motor activity	0	49.73563218	8
	bacterial‐type flagellum basal body	0	44.76206897	9
	bacterial‐type flagellum‐dependent cell motility	0	49.73563218	16
	chemotaxis	2.16E‐14	21.09996517	14
	structural molecule activity	3.74E‐08	41.44636015	5
	bacterial‐type flagellum organization	6.84E‐08	35.52545156	5
	bacterial‐type flagellum assembly	1.13E‐07	24.86781609	6
	bacterial‐type flagellum hook	7.45E‐07	49.73563218	4
	siderophore biosynthetic process	8.16E‐07	49.73563218	4
	bacterial‐type flagellum	1.59E‐06	33.15708812	4
	bacterial‐type flagellum basal body, rod	6.02E‐06	49.73563218	3
	extracellular region	3.50E‐05	15.54238506	5
	siderophore uptake transmembrane transporter activity	4.90E‐05	28.42036125	4
	signaling receptor activity	4.90E‐05	28.42036125	4
	signal transduction	9.76E‐05	11.47745358	6
	acid‐amino acid ligase activity	0.00012612	37.30172414	3
	protein‐glutamine glutaminase activity	0.00038634	49.73563218	2
	archaeal or bacterial‐type flagellum‐dependent cell motility	0.00100179	33.15708812	2
	protein secretion	0.00192411	9.042842215	4
	transmembrane signaling receptor activity	0.00259412	10.47065941	4
	plasma membrane	0.00904467	2.103248127	17

^a^ Clusters are referred to parentheses in Figure 1B.

### 3.4 Swimming Motility of Δ*ralT*

**Figure 4:**
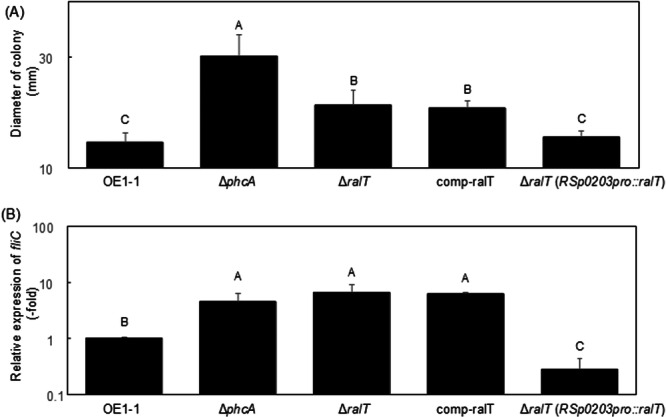
Swimming motility (A) of *Ralstonia pseudosolanacearum* strains and the *fliC* expression level (B) in strains according to a quantitative real‐time polymerase chain reaction analysis. (A) Strain OE1‐1, *phcA*‐deletion (Δ*phcA*), *ralA*‐deletion (Δ*ralA*), and *ralT*‐deletion (Δ*ralT*) mutants, transformant (comp‐ralT) with the *ralT* promoter and open reading frame, and transformant [Δ*ralT* (*RSp0203pro::ralT*)] with the *RSp0203* promoter fused to the *ralT* open reading frame were grown on quarter‐strength M63 medium containing 0.3% w/v agar. Fifteen technical replicates were prepared per group. Bars indicate standard errors. Means were analyzed for significant differences between *R. pseudosolanacearum* strains via an analysis of variance (ANOVA) followed by Tukey–Kramer's honestly significant difference test. Statistically significant differences are indicated by different lowercase letters (*α* = 0.05). (B) Strains were grown in quarter‐strength M63 medium until the optical density at 600 nm (OD_600_) reached 0.3 and then used for an RNA extraction. The *rpoD* transcript level was used as the internal standard to normalize all values and 2‐ΔΔCt for the calculation of *fliC* expression level. There were no significant differences in the *rpoD* transcript level among *R. pseudosolanacearum* strains. Experiments were conducted using five biological replicates. Bars indicate standard errors. Means were analyzed for significant differences between *R. pseudosolanacearum* strains via an ANOVA followed by Tukey–Kramer's honestly significant difference test. Statistically significant differences are indicated by different lowercase letters (*α* = 0.05).

Under culture conditions, flagella‐driven RSSC swimming motility is a growth‐phase dependent process that is negatively controlled by PhcA during the QS‐active state (Tans‐Kersten et al. 2001). Furthermore, the *ralA*‐deletion significantly enhanced the expression level of *fliC* included in *fli* operon genes, leading to a significant increase in swimming motility of the Δ*ralA* mutant (Mori et al. 2018b). A GO analysis indicated that GO terms related to bacterial motility, such as “chemotaxis,” “bacterial‐type flagellum basal body,” and “bacterial‐type flagellum‐dependent cell motility,” were enriched among negatively QS/Ral/p0599‐dependent genes. The transcriptome analysis showed that the deletion of *ralT* significantly enhanced the expression of *fli* operon genes, including *fliC* (Table S8). We examined the *in vitro* swimming motility of *R. pseudosolanacearum* strains grown in quarter‐strength M63 medium solidified with 0.25% w/v agar. Compared with strain OE1‐1, the Δ*ralT* mutant exhibited significantly enhanced swimming motility, similar to Δ*phcA* (*α* = 0.05; Figure 4A).

The swimming motility of the Δ*ralT* mutant transformed with the pUC18‐mini‐Tn*7*T‐GM‐based plasmid (pralT) carrying the native *ralT* promoter and open reading frame (comp‐ralT) was similar to that of Δ*ralT* (Figure 4A). Furthermore, comp‐ralT was also similar to Δ*ralT* in terms of *fliC* expression (Figure 4B). Thus, we hypothesized that *ralT* may not be expressed in comp‐ralT. A transcriptome analysis showed that the deletion of *phcA*, *ralA*, or *ralT* did not alter the expression level of *RSp0203* encoding acyl‐CoA thioester hydrolase‐related protein. Moreover, count values of *RSp0203* were similar level to those of *ralT* in strain OE1‐1 (Table S1). We then transformed Δ*ralT* with plasmid pRSp0203proralT (Table 1), which includes the *RSp0203* promoter fused to the *ralT* open reading frame, to generate the Δ*ralT* (*RSp0203pro::ralT*) transformant. Compared with Δ*ralT*, Δ*ralT* (*RSp0203pro::ralT*) had a significantly lower *fliC* expression level (Figure 4B). Furthermore, Δ*ralT* (*RSp0203pro::ralT*) exhibited significantly decreased swimming motility (relative to that of Δ*ralT*) (Figure 4A). These results suggest that RalT may repress *fli* operon expression, thereby suppressing the swimming motility of strain OE1‐1.

### 3.5 EPS I Production of Δ*ralT*

**Figure 5:**
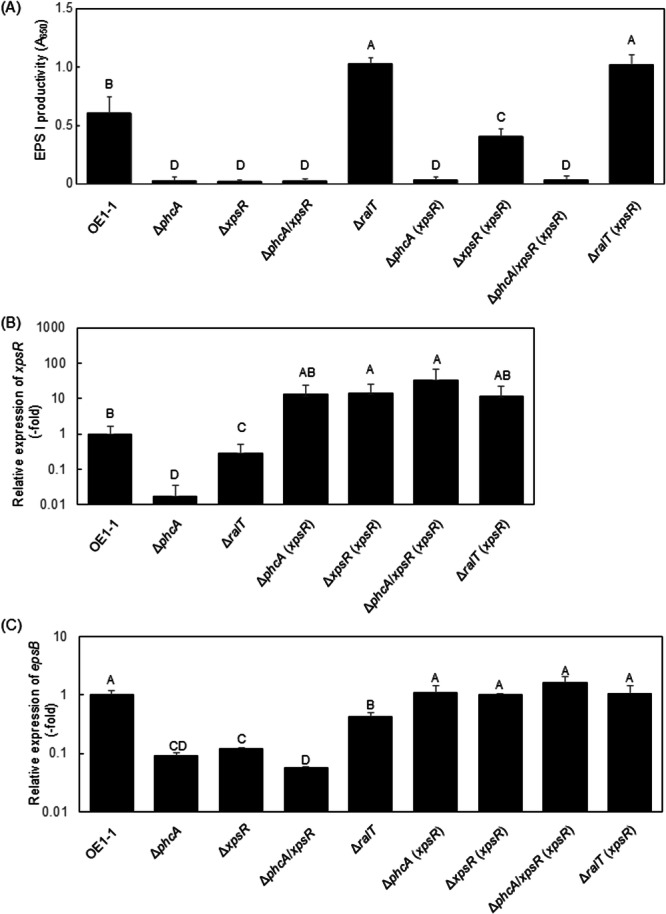
Major exopolysaccharide EPS I production (A) in *Ralstonia pseudosolanacearum* strains, and expression levels of *xpsR* (B) and *epsB* (C) in strains according to a quantitative real‐time polymerase chain reaction analysis. (A) Strain OE1‐1, *phcA*‐deletion (Δ*phcA*), *ralA*‐deletion (Δ*ralA*), *ralT*‐deletion (Δ*ralT*), *xpsR*‐deletion (Δ*xpsR*), and *phcA*/*xpsR*‐deletion (Δ*phcA*/*xpsR*) mutants, and Δ*phcA* (*xpsR*), Δ*xpsR* (*xpsR*), Δ*phcA*/*xpsR* (*xpsR*), and Δ*ralT* (*xpsR*) transformants, which were derived from Δ*phcA*, Δ*xpsR*, Δ*phcA*/*xpsR*, and Δ*ralT* transformed with the pHM1‐based plasmid pRSc0900proxpsR containing the constitutively active *RSc0900* promoter fused to *xpsR*, were incubated on quarter‐strength M63 medium solidified with 1.5% agar. The EPS I content of supernatants was quantified in an enzyme‐linked immunosorbent assay involving anti‐*R. solanacearum* EPS I antibodies. EPS I production was quantified according to the absorbance at 650 nm (A_650_). Twenty‐one technical replicates were prepared per group. Bars indicate standard errors. Means of EPS I production were analyzed for significant differences between strains via an analysis of variance (ANOVA) followed by Tukey–Kramer's honestly significant difference test. Statistically significant differences are indicated by different lowercase letters (*α* = 0.05). (B, C) Strains were grown in quarter‐strength M63 medium until the optical density at 600 nm (OD_600_) reached 0.3 and then used for an RNA extraction. The *rpoD* transcript level was used as the internal standard to normalize all values and 2‐ΔΔCt for the calculation of *epsB* expression level. There were no significant differences in the *rpoD* transcript level among *R. pseudosolanacearum* strains. Experiments were conducted using five biological replicates. Bars indicate standard errors. Means were analyzed for significant differences between strains via an ANOVA followed by Tukey–Kramer's honestly significant difference test. Statistically significant differences are indicated by different lowercase letters (*α* = 0.05).

During QS, activated PhcA induces the transcription of *xpsR*, with the encoded transcriptional regulator (XpsR) binding to the promoter of *eps* operon genes, including *epsB*, and increasing expression (XpsR‐regulated pathway; Garg et al. 2000). The *ralA*‐deletion leads to a significant decrease in EPS I production (Mori et al. 2018b). Furthermore, the transcriptome analysis showed that the deletion of *phcA* or *ralA* significantly reduces expression level of EPS I production‐related genes including *epsB* as well as *xpsR* (Table S9). To examine contribution RalT to EPS I production, we examined the *in vitro* EPS I production of *R. pseudosolanacearum* strains grown in quarter‐strength M63 medium solidified with 1.5% agar. The *xpsR*‐deletion led to a significant decrease in EPS I production, similar to the *phcA*‐deletion (*α* = 0.05; Figure 5A). Furthermore, the qRT‐PCR analysis showed that the *phcA*‐deletion led to a significant decrease in the expression level of *xpsR* (*α* = 0.05; Figure 5B). The expression level of *epsB* significantly decreased in Δ*phcA* at the similar level in Δ*xpsR*, compared to that in OE1‐1 (*α* = 0.05; Figure 5C). These results suggest that the PhcA/XpsR‐regulated pathway mainly contributes to the induction of EPS I production.

On the other hand, the Δ*ralT* mutant produced significantly more EPS I than strain OE1‐1 (*α* = 0.05; Figure 5 A), suggesting that RalT is involved in the negative regulation of EPS I production. However, an RNA‐seq analysis showed that the deletion of *ralT* led to a significant decrease in the expression levels of *eps* genes including *epsB* as well as *xpsR* (Table S9). In addition, though the qRT‐PCR analysis showed that the expression levels of *xpsR* and *epsB* in Δ*ralT* is significantly lower than those in strain OE1‐1, Δ*ralT* significantly exhibited high expression levels of *xpsR* and *epsB* than Δ*phcA* (*α* = 0.05; Figure 5B,C). Thus, we hypothesized that the factor(s) other than the XpsR may be positively regulated by PhcA but not XpsR and be required for the induction of EPS I.

To assess this hypothesis, we generated Δ*xpsR* and Δ*phcA*/*xpsR* mutants from strain OE1‐1 and Δ*phcA*, respectively. RNA‐seq data indicated that the deletion of *phcA*, *ralA*, or *ralT* did not affect expression of *RSc0900* encoding signal peptide protein of which function is unknown. In addition, count values of *RSc0900* were similar level to those of *xpsR* in strain OE1‐1 (Table S1). We then transformed the Δ*phcA*, Δ*xpsR*, Δ*phcA*/*xpsR*, and Δ*ralT* mutants with the pHM1‐based plasmid pRSc0900proxpsR, which contains *xpsR* fused to the constitutively activated *RSc0900* promoter, thereby generating the Δ*phcA* (*xpsR*) Δ*xpsR* (*xpsR*), Δ*phcA*/*xpsR* (*xpsR*), and Δ*ralT* (*xpsR*) mutants, respectively. The qRT‐PCR analysis showed that all transformants expressed similar expression levels of *xpsR* (Figure 5B), leading to the similar expression level of *epsB* in these mutants, compared to that in strain OE1‐1 (Figure 5 C). We then assayed EPS I production in the transformants. The Δ*phcA*/*xpsR* mutant produced significantly less EPS I than strain OE1‐1, similar to the Δ*xpsR* and Δ*phcA* mutants (*α* = 0.05; Figure 5A). The Δ*xpsR* (*xpsR*) mutant had significantly more EPS I than the Δ*xpsR* mutant. However, the induction of *xpsR* expression independent of PhcA did not enhance the production of EPS I in the Δ*phcA*, Δ*phcA*/*xpsR*, and Δ*ralT* mutants. These results suggest that the XpsR‐independent factor(s) is positively regulated by PhcA and are required for EPS I production. The *ralT*‐deletion led to a decrease in *xpsR* expression levels (Figure 5B) and an increase in EPS I production (Figure 5A). It is thus thought that RalT may contribute to the negative regulation of the XpsR‐independent factor(s) as well as the positive regulation of *xpsR*.

### 3.6 Virulence of Δ*ralT*

**Figure 6:**
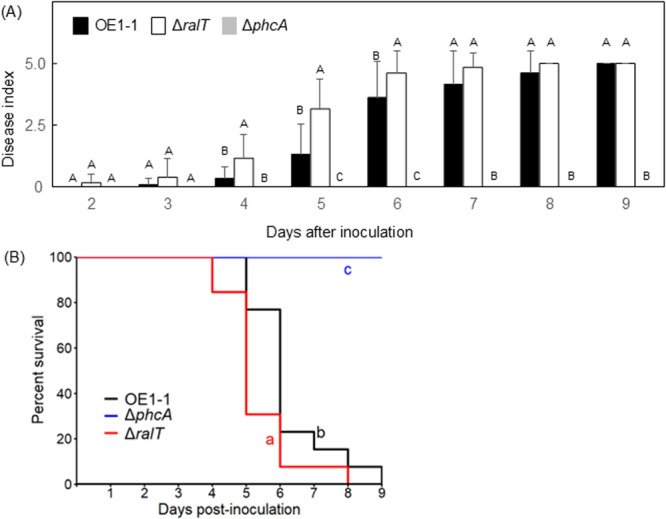
Virulence (A) of *Ralstonia pseudosolanacearum* strains on tomato plants and survival rate of the tomato plants (B). (A) Eighteen‐day‐old tomato plants were inoculated with *R. pseudosolanacearum* strain OE1‐1 or the *phcA*‐deletion (Δ*phcA*) and *ralT*‐deletion (Δ*ralT*) mutants. Plants were rated according to the following disease index scale: 0, no wilting; 1, 1%–25% wilting; 2, 26%–50% wilting; 3, 51%–75% wilting; 4, 76%–99% wilting; 5, dead. For each bacterial strain, 20 technical replicates were tested. Bars indicate standard errors. Means were analyzed for significant differences between *R. pseudosolanacearum* strains via an analysis of variance followed by Tukey–Kramer's honestly significant difference test. Statistically significant differences are indicated by different lowercase letters (*α* = 0.05). (B) Disease scoring was transformed into binary data, with a disease index below 3 corresponding to 0 and a disease index equal to or higher than 3 corresponding to 1. Log‐rank pairwise tests were used to determine significant difference between strains. A *p*‐value smaller than 0.05 was considered significant, indicating that the Ho hypothesis of similarity of the survival experience of the tested strains can be rejected. Statistical analyses were done with RStudio.

The Δ*ralA* loses its virulence (Kai et al. 2014) as well as the Δ*phcA* (Hikichi et al. 2017). To investigate the effects of RalT on strain OE1‐1 virulence, we inoculated 18‐day‐old tomato plants with *R. pseudosolanacearum* strains according to the root‐dip method (Hayashi et al. 2019a) and then examined disease development. For the tomato plants inoculated with strain OE1‐1, wilt symptoms were detectable 3 days after inoculation (DAI), and all plants were dead by 9 DAI (Figure 6A). Tomato plants inoculated with Δ*phcA* did not exhibit any wilt symptom. Notably, the tomato plants inoculated with Δ*ralT* mutant had a much higher disease index, compared to those with OE1‐1. Furthermore, the Kaplan–Meier survival analysis of tomato plants showed that the tomato plants inoculated with Δ*ralT* mutant had a significantly lower survival rate, compared to those with OE1‐1 (Figure 6B).

## 4 Discussion

The members of the TetR/AcrR family of transcriptional regulators harbor a diverse ligand‐binding domain that recognizes the same series of compounds as the transporters they regulate (Colclough et al. 2019; Routh et al. 2009). RalT has a C‐terminal ligand‐binding domain as well as an N‐terminal helix‐turn‐helix motif, similar to TetR/AcrR family transcriptional regulators (Figure 3A). Furthermore, the TetR/AcrR family transcriptional regulators usually bind to incomplete palindromic sequences in the upstream region of their own gene and repress expression (Deng et al. 2013). In the current study, the putative *ralT* promoter region was revealed to contain a palindromic sequence (Figure 3B). The *ralA*‐deletion reduced the expression levels of *RSp0600* as well as *ralT* (Figure 3C). A GUS activity assay showed that RalT repressed the expression of *ralT* as well as *RSp0600* (Figure 3D). Therefore, *ralT* encodes a novel TetR/AcrR family transcriptional regulator.

QS is tightly linked to the production of various types of chemical compounds. Moreover, the positive feedback regulation of QS is important for the rapid activation of QS and the synchronization of QS‐dependent gene regulation (Engebrecht and Silverman 1984; More et al. 1996; Ng and Bassler 2009; Schaefer et al. 1996). The production of an aryl‐furanone secondary metabolite, ralfuranone, is induced in the QS‐active state (Kai 2023; Kai et al. 2014, 2016) and is involved in the positive QS feedback regulation by influencing the regulation of QS/Ral‐dependent genes (Mori et al. 2018b). Results in this study showed that ralfuranone, but not QS, participates in the positive regulation of *ralT* expression (Figure 2). Of the positively and negatively QS/Ral‐dependent genes, 89.4% and 44.6% had expression levels that decreased and increased significantly, respectively, after the deletion of *ralT* (Figure 1A,B). In addition, QS/Ral/p0599‐dependent gene expression levels were positively correlated between Δ*ralT* and Δ*phcA* or Δ*ralA* (Figure 1C,D). These results indicate the contribution of RalT on the regulation of some QS/Ral‐dependent genes (Figure S1).

In many bacterial species, TetR/AcrR family transcriptional regulators act as chemical sensors that monitor the cellular environment to regulate a wide range of cellular activities, properties, and components, including osmotic stress responses, homeostasis, biosynthesis of antibiotics, multidrug resistance, efflux pumps, enzymes implicated in different catabolic pathways, and virulence (Ahn et al. 2012; Colclough et al. 2019; Deng et al. 2013; Ramos et al. 2005; Routh et al. 2009). In the present study, the deletion of *ralA* but not *phcA* resulted in significantly reduced expression of *ralT* (Figure 2). RalT repressed the expression of *ralT* (Figure 3D) and induced the expression of *ralA* and *ralD* encoding ralfuranone biosynthesis‐related proteins (Table S6), suggesting that ralfuranone biosynthesis‐related genes are regulated via positive feedback through RalT (Figure S1). Thus, the regulation of QS/Ral/p0599‐dependent genes may be fine‐tuned through RalT.

Flagella biogenesis in RSSC is negatively regulated during the QS‐activation state because of activated PhcA (Tans‐Kersten et al. 2001). Furthermore, ralfuranone deficiency enhances swimming motility (Mori et al. 2018b). The results of the current study showed that RalT negatively regulates the expression of swimming motility‐related genes, including *fliC*, which were included among the ralfuranone‐mediated negative QS‐genes (Table S8). The transformation of Δ*ralT* with pRSp0203proralT (with a constitutively activated promoter) resulted in a significant decrease in swimming motility to a level that was similar to that of strain OE1‐1 (Figure 4). Accordingly, RalT may directly repress the expression of swimming motility‐related genes along with PhcA (Figure S1), thereby decreasing swimming motility.

In the QS‐active state, PhcA binds to the promoter of *xpsR*, which encodes the transcriptional regulator XpsR, to enhance expression (Garg et al. 2000; Genin and Denny 2012). XpsR positively regulates *eps* operon genes involved in the production and secretion of EPS I, resulting in enhanced EPS I production. The requirement of XpsR for EPS I production (Figure 5A) suggests that the XpsR‐regulated pathway mainly contributes to the induction of EPS I production. The experiment involving the QS‐independent and ectopic expression of *xpsR* suggests that XpsR‐independent factor(s), which is positively regulated by PhcA, is required for EPS I production. It is thought that RalT contributes to both positive regulation of *xpsR* and the negative regulation of the XpsR‐independent factor(s) (Figure 5B). Because *ralT* was regulated independently of PhcA (Figure 2), RalT contributes to the sophisticated regulation of EPS I production, which is positively regulated through PhcA and XpsR.

RalT positively regulated many virulence‐related genes such as *lecM*, *cbhA*, and *egl* of QS/Ral/p0599‐dependent genes (Table S6). However, tomato plants inoculated with Δ*ralT* wilted faster than those inoculated with strain OE1‐1 (Figure 6), suggesting RalT negatively regulates strain OE1‐1 virulence. In RSSC, a deficiency in the type III secretion machinery leads to a loss of virulence (Genin and Denny 2012; Hikichi et al. 2017). Although genes encoding the type III secretion machinery components and the type III effectors were included among the negative QS/Ral‐dependent genes but not negatively QS/Ral/p0599‐dependent genes (cluster B‐ IV), their regulation was independent of RalT. However, EPS I, which is a major virulence factor of RSSC (Genin and Denny 2012), contributes to the positive QS feedback regulation separately from ralfuranone (Hayashi et al. 2019b). In the present study, deleting *ralT* led to enhanced EPS I production (Figure 5A). We thus speculated that RalT may negatively regulate strain OE1‐1 virulence by decreasing EPS I production.

VsrAD and VsrBC two‐component sensor histidine kinase/response regulator systems affect the regulation of flagella biogenesis (Genin and Denny 2012). Furthermore, PhcA and the VsrAD two‐component system are necessary to fully activate *xpsR* transcription and positively regulate the *eps* operon, thereby enhancing EPS I production (Garg et al. 2000; Genin and Denny 2012). Both XpsR and the VsrC bind to the promoters of *eps* operon genes, including *epsB*, to enhance expression (Garg et al. 2000). Schneider et al. (2009) reported that VsrAD functions upstream of PhcA and contributes to ralfuranone biosynthesis. Therefore, QS‐dependent phenotypes, such as swimming motility and EPS I production, are regulated through RalT along with VsrAD and VsrBC two‐component systems. Results in this study indicate that RalT negatively regulated *ralT* (Figure 3D) and is involved in the positive feedback regulation of ralfuranone production‐related genes (Table S6). Considered together, these findings indicate that RalT‐mediated gene regulation may lead to the comprehensive regulation of RSSC virulence, with QS representing a critical regulatory system. In terms of future research, *in vitro* experiments using purified proteins and signal transduction assays will be important for elucidating the QS mechanisms governing RalT‐mediated gene regulation. Because inhibiting QS via the application of phc quorum sensing inhibitors (PQIs) can control bacterial wilt caused by strain OE1‐1 (Yoshihara et al. 2020), comprehensively and precisely characterizing the QS mechanism, including RalT‐mediated gene regulation, may reveal molecular targets appropriate for improving disease control measures.

## Author Contributions

**Tatsuya Ueyama:** funding acquisition, investigation, methodology, validation and visualization. **Masayuki Tsuzuki:** conceptualization, funding acquisition, investigation, methodology, validation, visualization, writing – review and editing. **Sora Tateda:** funding acquisition, investigation, methodology, validation and visualization. **Yuki Terazawa:** investigation, methodology, validation and visualization. **Aoi Ikeuchi:** investigation, methodology, validation and visualization. **Akinori Kiba:** conceptualization. **Kouhei Ohnishi:** conceptualization. **Yasufumi Hikichi:** conceptualization, funding acquisition, writing – original draft.

## Ethics Statement

The authors have nothing to report.

## Conflicts of Interest

The authors declare no conflicts of interest.

## Supporting information


**FIGURE S1:** Predicted regulation of QS/Ral/p0599‐dependent genes via RalT in *Ralstonia pseudosolanacearum* strain OE1‐1.

**TABLE S1:** RNA‐sequencing data for transcripts of genes in *Ralstonia pseudosolanacearum* strain OE1‐1, and *phcA*‐deletion (Δ*phcA*), *ralA*‐deletion (Δ*ralA*) and *ralT*‐deletion (Δ*ralT*) mutants, grown in quarter‐strength M63 medium, and predicted function of proteins encoded the genes.

**TABLE S2:** RNA‐sequencing data for transcripts of QS‐dependent genes in *Ralstonia pseudosolanacearum* strain OE1‐1, and *phcA*‐deletion (Δ*phcA*), *ralA*‐deletion (Δ*ralA*) and *ralT*‐deletion (Δ*ralT*) mutants, grown in quarter‐strength M63 medium, and predicted function of proteins encoded the genes.

**TABLE S3:** RNA‐sequencing data for transcripts of QS/RalA‐dependent genes in *Ralstonia pseudosolanacearum* strain OE1‐1, and *phcA*‐deletion (Δ*phcA*), *ralA*‐deletion (Δ*ralA*) and *ralT*‐deletion (Δ*ralT*) mutants, grown in quarter‐strength M63 medium, and predicted function of proteins encoded the genes.

**TABLE S4:** RNA‐sequencing data for transcripts of genes whose expression levels were reduced in the *ralA* deletion (Δ*ralA*) mutant but not the *phcA* deletion (Δ*phcA*), compared to *Ralstonia pseudosolanacearum* strain OE1‐1, and predicted function of proteins encoded the genes.

**TABLE S5:** RNA‐sequencing data for quorum sensing‐related genes in *Ralstonia pseudosolanacearum* strain OE1‐1, and *phcA*‐deletion (Δ*phcA*), *ralA*‐deletion (Δ*ralA*) and *ralT*‐deletion (Δ*ralT*) mutants, grown in quarter‐strength M63 medium, and predicted function of proteins encoded the genes.

**TABLE S6:** RNA‐sequencing data for transcripts of p0599‐dependent genes in *Ralstonia pseudosolanacearum* strain OE1‐1, and *phcA*‐deletion (Δ*phcA*), *ralA*‐deletion (Δ*ralA*) and *ralT*‐deletion (Δ*ralT*) mutants, grown in quarter‐strength M63 medium, and predicted function of proteins encoded the genes.

**TABLE S7:** RNA‐sequencing data for transcripts of QS/Ral/p0599‐dependent genes in *Ralstonia pseudosolanacearum* strain OE1‐1, and *phcA*‐deletion (Δ*phcA*), *ralA*‐deletion (Δ*ralA*) and *ralT*‐deletion (Δ*ralT*) mutants, grown in quarter‐strength M63 medium, and predicted function of proteins encoded the genes.

**TABLE S8:** RNA‐sequencing data for transcripts of flagellar motility‐related genes in *Ralstonia pseudosolanacearum* strain OE1‐1, and *phcA*‐deletion (Δ*phcA*), *ralA*‐deletion (Δ*ralA*) and *ralT*‐deletion (Δ*ralT*) mutants, grown in quarter‐strength M63 medium, and predicted function of proteins encoded the genes.

**TABLE S9:** RNA‐sequencing data for transcripts of EPS I production‐related genes in *Ralstonia pseudosolanacearum* strain OE1‐1, and *phcA*‐deletion (Δ*phcA*), *ralA*‐deletion (Δ*ralA*) and *ralT*‐deletion (Δ*ralT*) mutants, grown in quarter‐strength M63 medium, and predicted function of proteins encoded the genes.

## Data Availability

Data supporting the findings of this study are available from the corresponding author upon reasonable request. RNA‐seq data are available in the NCBI SRA repository (https://www.ncbi.nlm.nih.gov/sra/) under accession codes DRR493625, DRR438005, DRR438006, DRR438007, and DRR493446 (WT: OE1‐1); DRR438008, DRR438009, DRR438010, DRR450853, DRR450854, and DRR493614 (Δ*phcA*); PRJNA1244431 (Δ*ralT*).
