# Supplementary figures and images for: Contribution of a Novel TetR/AcrR Family Transcriptional Regulator RalT of Ralstonia pseudosolanacearum Strain OE1‐1 to the Fine‐Tuning of Its Virulence
Source: Microbiologyopen. 2026 Feb 22;15(1):e70229. doi: 10.1002/mbo3.70229 (PMC12928067; doi:10.1002/mbo3.70229)

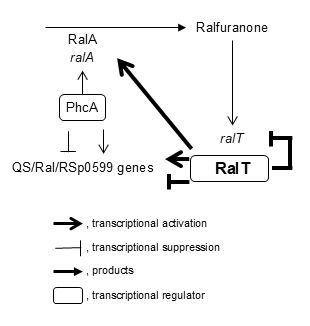

Supplement: Supplementary file 1 — FIGURE S1: Predicted regulation of QS/Ral/p0599‐dependent genes via RalT in Ralstonia pseudosolanacearum strain OE1‐1. [file MBO3-15-e70229-s009.tif]
